# Supplementary material for: Ventral tegmental area astrocytes orchestrate avoidance and approach behavior
Source: Nat Commun. 2019 Mar 29;10:1455. doi: 10.1038/s41467-019-09131-y (PMC6440962; doi:10.1038/s41467-019-09131-y)
Supplement: Supplementary file 1 — Supplementary Information [file 41467_2019_9131_MOESM1_ESM.pdf]

SUPPLEMENTARY FIGURES

Supplementary Figure 1. VTA Astrocytes are sensitive to glutamate via AMPA receptor activation.

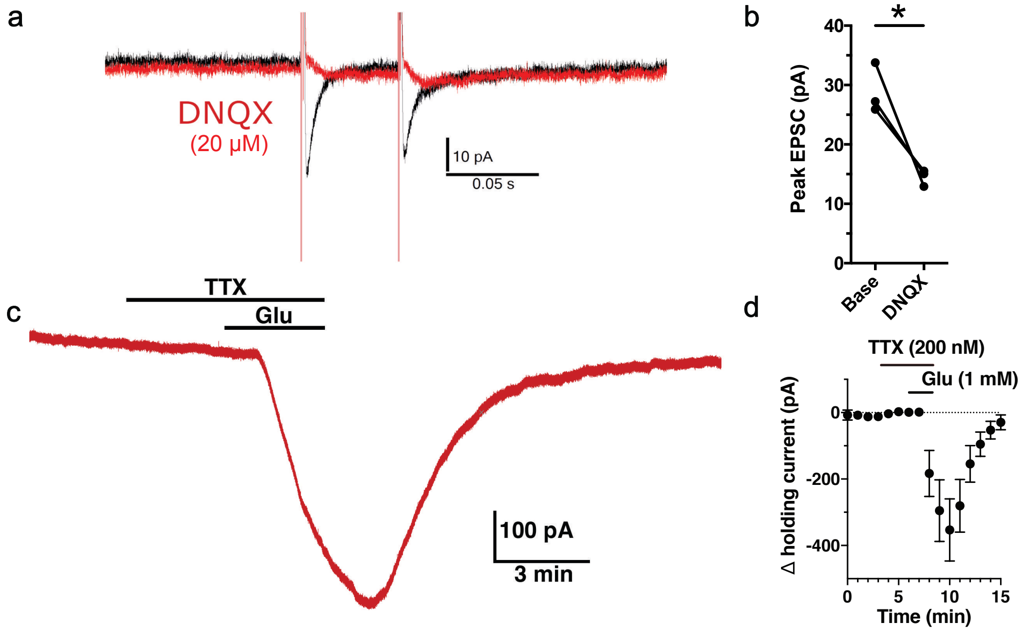

(a) Electrical stimulation during a whole-cell voltage clamp astrocyte recording evokes fast inward current responses (black traces). Bath application of the AMPA receptor blocker, DNQX (20 µM), blocks the response to electrical stimulation (red trace). **(b)** Summary data (paired t-test $t_{(2)} = 4.473$, $P = 0.047$, $n = 3$ cells from 2 mice). **(c)** Bath application of glutamate (1 mM) in the presence of TTX (200 nM) during a whole-cell recording of an astrocyte elicits a large inward current, indicating the presence of glutamate receptors on the recorded astrocyte. **(d)** Summary data (Repeated Measures ANOVA $t_{(6)} = 4.700$, $P = 0.003$, $n = 7$ cells from 3 mice, *: $P < 0.05$). Error bars indicate ± SEM.

Supplementary Figure 2. Channelrhodopsin is specifically expressed in astrocytes, not in neurons.

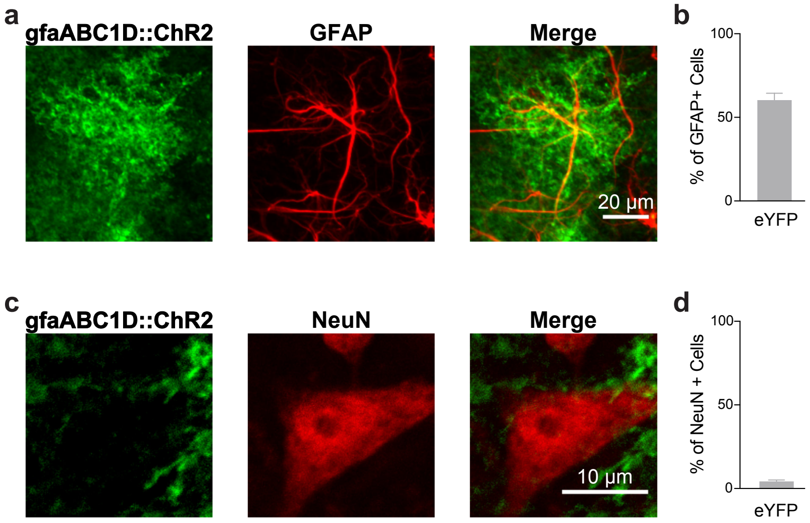

(a) Immunocytochemistry image showing a cell expressing both gfaABC1D-eYFP (green) and GFAP (red). On the right are the two images merged together. **(b)** Quantification of the percentage of GFAP positive cells expressing eYFP (n = 4 mice). **(c)** Image showing a NeuN positive cell (red) surrounded by eYFP (green). **(d)** Quantification of the percentage of NeuN positive cells expressing eYFP (n = 3 mice) Error bars indicate ± SEM.

Supplementary Figure 3. ChR2-mediated currents in VTA astrocytes possess properties similar to electrical stimulation.

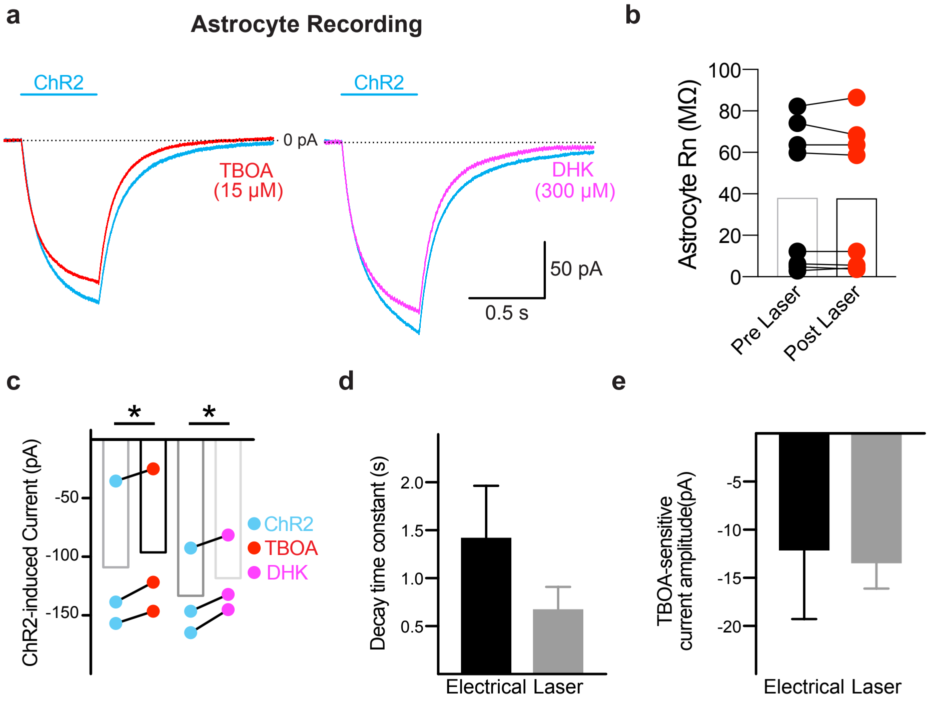

(a) Whole-cell recording from a VTA astrocyte showing a ChR2-mediated current (blue; no laser = 0.8325 ± 0.6587 pA, ChR2 = -87.06 ± 19.56 pA; paired t-test $t_{(8)} = 4.437$, P = 0.002, n = 9 cells, 4 mice) and the current remaining after application of 15 µm TFB-TBOA (left red) or 300 µm DHK (right magenta). **(b)** Summary of the astrocyte input resistance before and after stimulation of ChR2 in VTA astrocytes, indicating astrocytes remain healthy following photoactivation. **(c)** Summary of the ChR2 current produced in VTA astrocytes before (blue) and after (red) addition of TFB-TBOA (paired t-test $t_{(2)} = 5.607$, P = 0.03, n = 3 cells) or after (magenta) addition of DHK (paired t-test $t_{(2)} = 6.051$, P = 0.02, n = 3 cells, 2 mice). **(d)** Decay time constant (Tau) for the TBOA-sensitive current elicited by electrical stimulation of the slice (black bar) and ChR2 laser stimulation (gray bar) of the recorded astrocyte (unpaired t-test $t_{(6)} = 0.3568$, P = 0.35, n = 3, n = 5 cells respectively, 2 mice). **(e)** Peak amplitudes of the TFB-TBOA-sensitive currents (unpaired t-test $t_{(6)} = 0.1386$, P = 0.89, n = 3 and n =5 cells respectively, 2 mice). **: P < 0.01, *: P < 0.05; Error bars indicate ± SEM.

Supplementary Figure 4. Identification of VTA neurons.

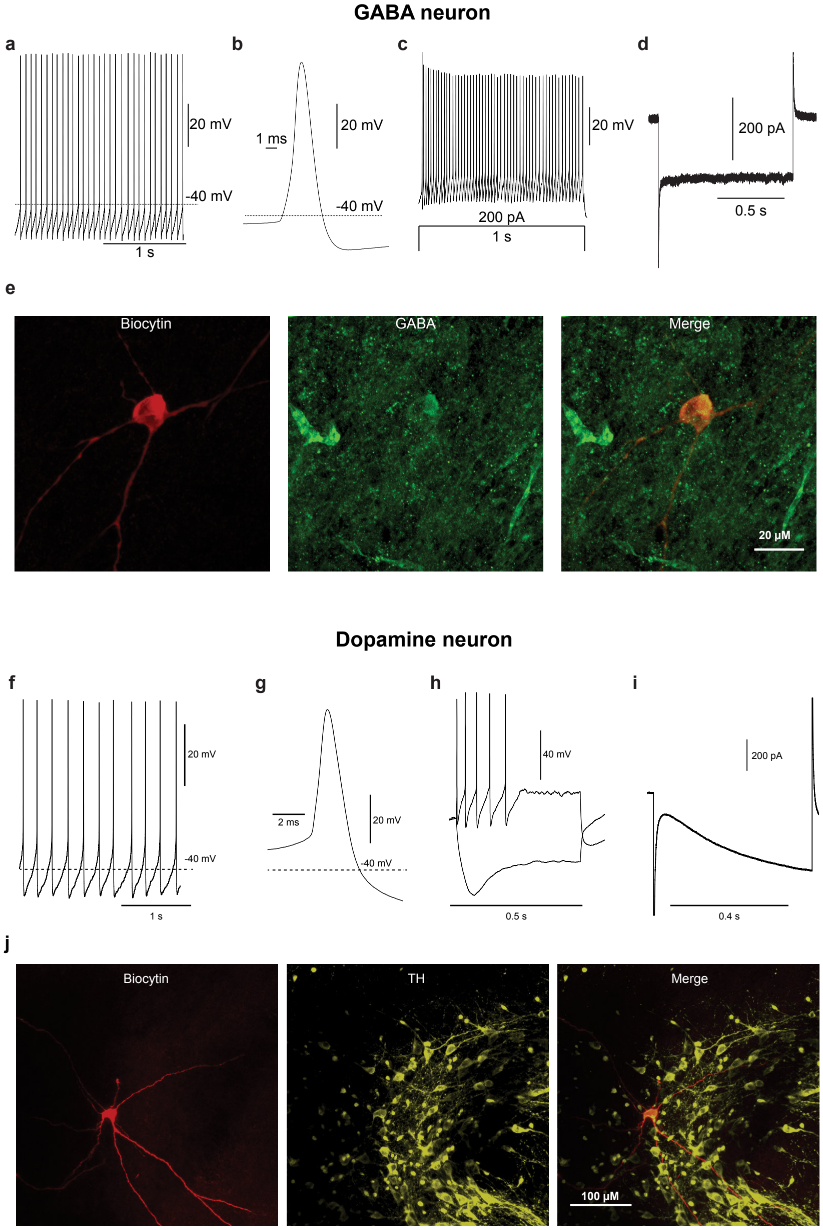

Whole-cell current-clamp recording from a GABA (a-e) neuron and dopamine neuron (f-i). (**a, b**) One of the action potentials from (a) was expanded to emphasize the action potential half width (average action potential half width = 0.99 ms). (**c**) Recording showing no spike frequency adaptation in response to a 200 pA current injection. **(d)** Voltage clamp recording showing lack of I_h in response to hyperpolarizing the cell from -60 mV to -120 mV for 1 second (average I_h = -37.9 pA). (**e**) Immunohistochemistry images taken with a 40X objective showing a biocytin filled cell visualized using Alexa 594 (red) and GABA visualized using Alexa 488 (green). On the right is a merged image to show co-localization between the two fluorophores. **(f, g)** One of the action potentials from (f) was expanded to emphasize the wide action potential half width (average half width = 3.37 ms) . **(h)** Example recording showing spike frequency adaptation in response to a 200 pA current injection and a characteristic sag in response to hyperpolarizing current injection (h; -200pA). **(i)** Developing I_h due to voltage clamp step (average I_h = -156.4 pA). **(j)** Immunohistochemistry images (20X objective) showing a biocytin-filled cell (red) with overlapping tyrosine hydroxylase staining (yellow). On the right is a merged image showing co-localization of the two fluorophores.

Supplementary Figure 5. The intrinsic properties of VTA neurons are not altered by removing GLT-1 from astrocytes or by photoactivation of astrocytes.

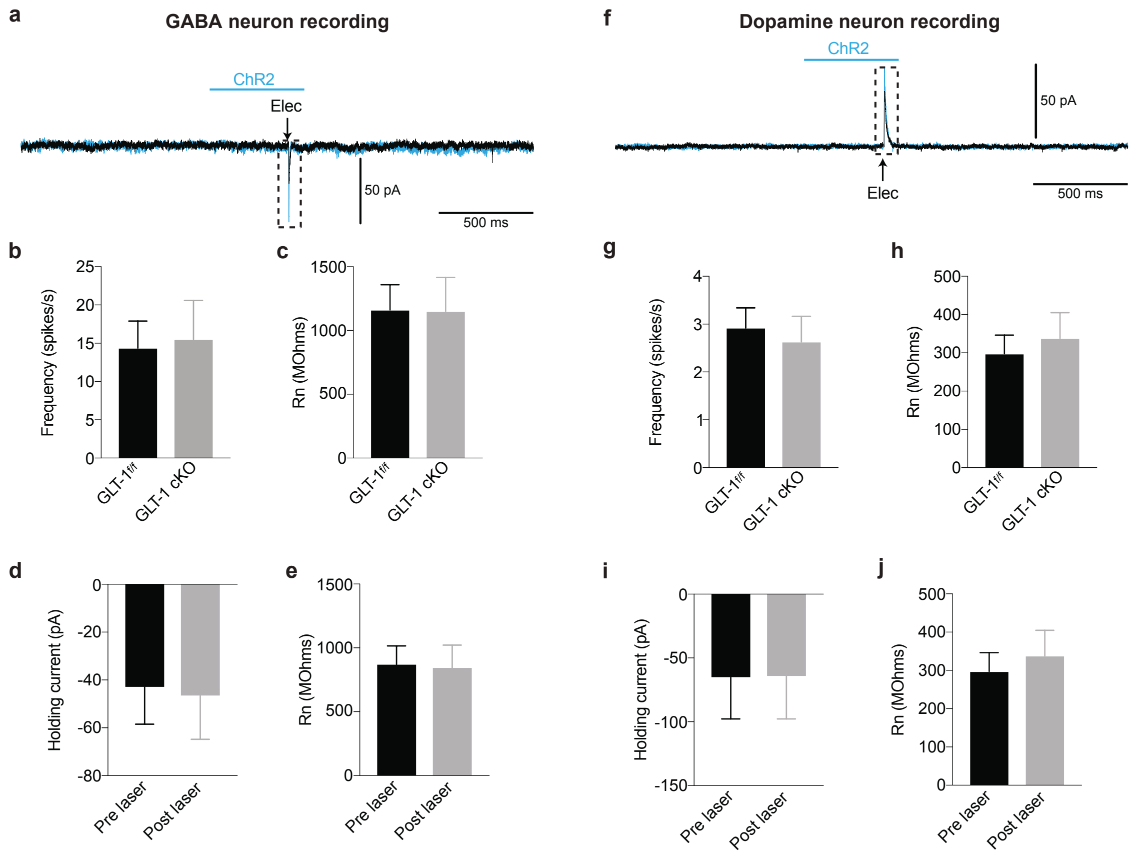

(a) Recording from a GABA neuron where astrocyte activation increases the EPSC. Summarized data showing there is no difference in **(b)** firing frequency (unpaired t-test $t_{(8)}$ = 0.1831, P = 0.85, GLT-1$^{f/f}$ n = 5 cells, 3 mice; GLT-1 cKO n = 5 cells, 2 mice) or **(c)** input resistance (unpaired t-test $t_{(12)}$ = 0.0282, P = 0.97, GLT-1$^{f/f}$ n = 6, 3 mice; GLT-1 cKO n = 8 cells, 2 mice) between GLT-1$^{f/f}$ or GLT-1 cKO mice. Summarized data from GABA neuron showing there is no change in the **(d)** holding current (paired t-test $t_{(8)}$ = 1.166, P = 0.27, n = 9 cells, 4 mice) or **(e)** input resistance (paired t-test $t_{(8)}$ = 0.2121, P = 0.83, n = 9 cells, 4 mice) before and after astrocyte activation. **(f)** Recording from a dopamine neuron where astrocyte activation increases the IPSC. Summarized data from dopamine cells showing there is no difference in **(g)** firing frequency (unpaired t-test $t_{(11)}$ = 0.4171, P = 0.68, GLT-1$^{f/f}$ n = 6 cells, 2 mice; GLT-1 cKO n = 7 cells, 2 mice) or **(h)** input resistance (unpaired t-test $t_{(13)}$ = 0.4818, P = 0.63, GLT-1$^{f/f}$ n = 7 cells, 2 mice; GLT-1 cKO n = 8 cells, 2 mice) between GLT-1$^{f/f}$ or GLT-1 cKO mice. Summarized data from dopamine neuron showing there is no change in the **(i)** holding current (paired t-test $t_{(5)}$ = 0.5782, P = 0.58, n = 6 cells, 3 mice) or **(j)** input resistance (paired t-test $t_{(5)}$ = 1.763, P = 0.13, n = 6 cells, 3 mice) before and after astrocyte activation. Error bars indicate ± SEM.

Supplementary Figure 6. Loss of GLT-1 expression from VTA astrocytes.

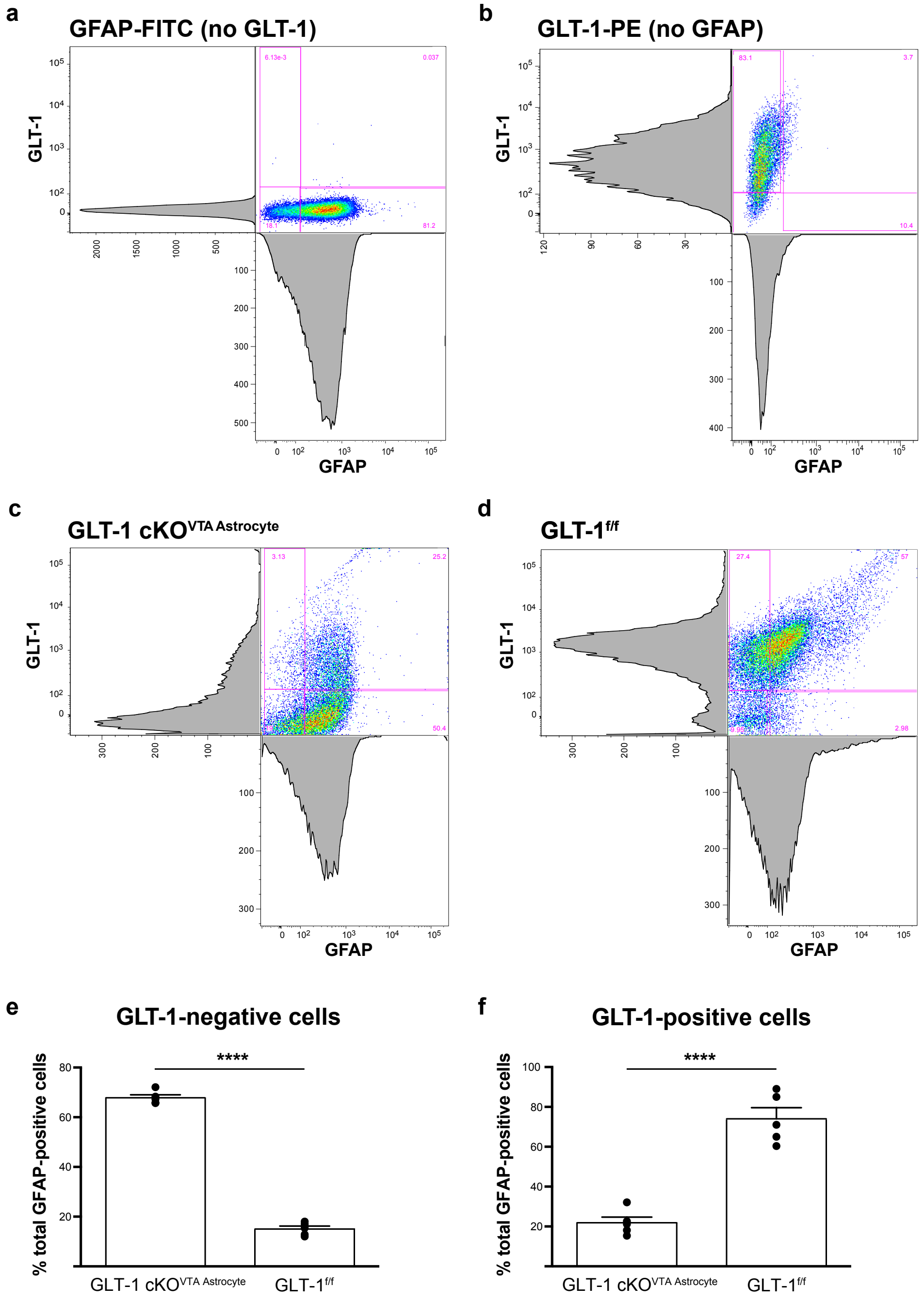

(a-d) Flow cytometry color dot-plots and histograms for GLT-1 (y-axis) and GFAP (x-axis) fluorescence intensities. **(a)** Control plot with no GLT-1 staining. **(b)** Control plot with no GFAP staining. **(c)** Flow cytometry from a GLT-1 cKO$^{VTA\ Astrocyte}$ (GLT-1$^{f/f}$, gfaABC1D::Cre^{VTA}) mouse with GLT-1 histogram peak indicating a shift toward GLT-1 negative cells relative to a GLT-1$^{f/f}$ mouse (d). The histogram peak for GFAP is not different from the control histogram in (a), indicating the specificity of the knockout. **(d)** GLT-1 and GFAP histograms from a GLT-1$^{f/f}$ mouse illustrates a large proportion of GLT-1 and GFAP positive cells (upper right quadrant) that is significantly reduced in the GLT-1 cKO$^{VTA\ Astrocyte}$ mouse (upper right quadrant in panel c). **(e, f)** Summary data indicating efficiency (e; GLT-1 negative cells: 67.84 ± 1.17% GLT-1 cKO$^{VTA\ Astrocyte}$; 15.04 ± 1.16% GLT-1$^{f/f}$; unpaired t-test $t_{(8)} = 32.02$, $P < 0.0001$, n = 5 mice each group) and specificity (f; GLT-1 positive cells: 21.88 ± 2.84% GLT-1 cKO$^{VTA\ Astrocyte}$; 74.08 ± 5.57% GLT-1$^{f/f}$; unpaired t-test $t_{(8)} = 8.345$, $P < 0.0001$, n = 5 mice each group) of the conditional knockout. ****: $P < 0.0001$; Error bars indicate ± SEM

Supplementary Figure 7. Loss of GLT-1 increases the decay kinetics and decreases maximum current generated on VTA GABA neurons.

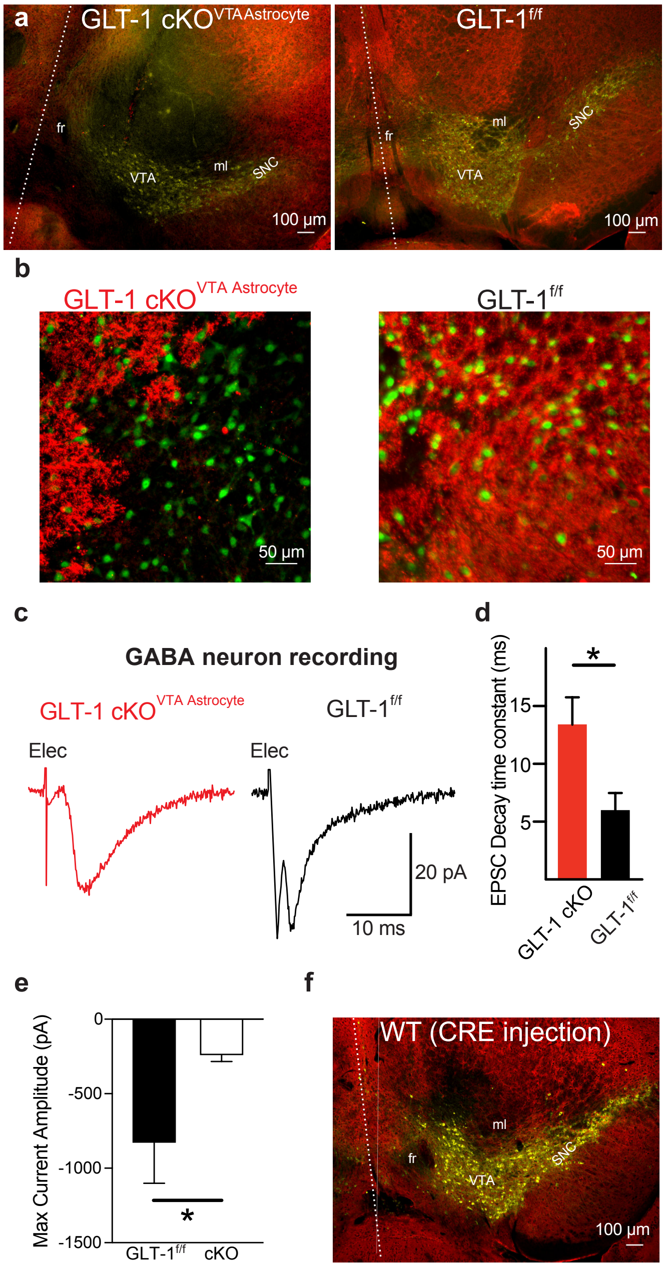

(a) Images (4X objective) illustrating loss of GLT-1 expression (red) in the VTA (GLT-1 cKO$^{VTA\ Astrocyte}$, left) versus a mouse with a control injection (GLT-1$^{f/f}$, right). For both (a) and (b) yellow is TH expression. **(b)** Image (20 X) showing NeuN positive cells (green) with GLT-1 staining (red; left image is edge of injection site). **(c)** Recordings from GABA neuron EPSC's in a GLT-1 cKO$^{VTA\ Astrocyte}$ (red) and a GLT-1$^{f/f}$ (black) mouse. **(d)** GLT-1 cKO$^{VTA\ Astrocyte}$ mice (red trace) have a slower decay time constant of the EPSCs compared to GLT-1$^{f/f}$ mice (black trace; unpaired t-test $t_{(9)} = 2.531$, $P = 0.03$, $n = 6$ and $n = 5$ cells respectively, 2 mice each). **(e)** Summarized data showing a difference in the maximum current generated when 200 µM glutamate is uncaged onto GABA neurons recorded from GLT-1$^{f/f}$ and GLT-1 cKO mice (unpaired t-test $t_{(15)} = 2.548$, $P = 0.022$, $n = 10$ and $n = 7$ cells respectively, 3 mice each). **(f)** 4X image illustrating normal GLT-1 expression (red) when Cre is driven in a wild type (WT) mouse (GLT-1$^{+/+}$, gfaABC1D::Cre^{VTA}). fr = fasciculus retroflexus; ml = medial lemniscus; SNC = substantia nigra pars compacta; VTA = ventral tegmental area. Dashed line indicates midline. For all images dorsal is up. *: $P < 0.05$; Error bars indicate ± SEM.

Supplementary Figure 8. Astrocyte activation does not directly affect GABA or dopamine neurons.

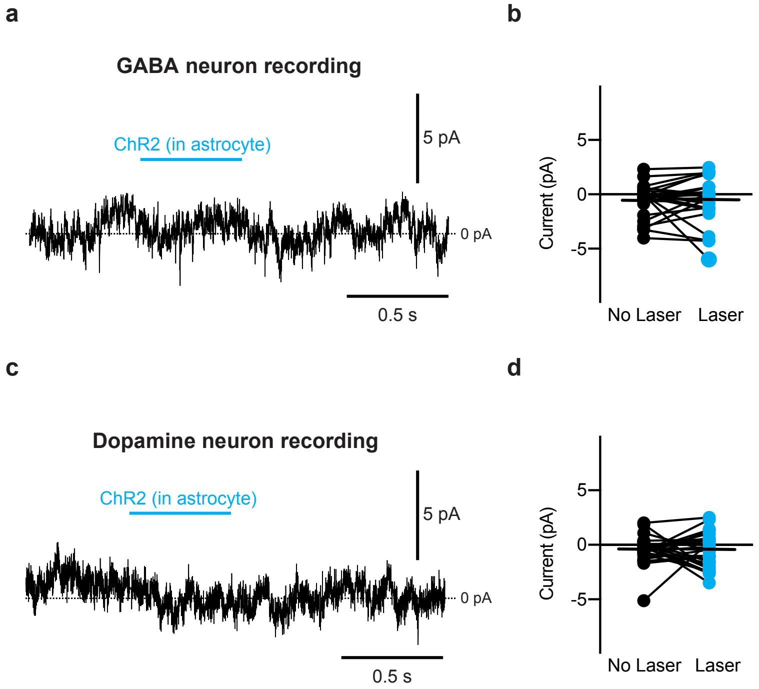

(**a**) Recording from a VTA GABA and **(c)** dopamine neuron while activating astrocytes expressing ChR2 (500 ms constant light pulse). Summary data comparing the average holding current 100 ms before astrocyte stimulation with the last 100 ms of astrocyte stimulation for recordings from **(b)** GABA (paired t-test $t_{(24)}$ = 0.215, P = 0.98, n = 25 cells, 9 mice) and **(d)** dopamine neurons (paired t-test $t_{(28)}$ = 0.2414, P = 0.811, n = 25 cells, 11 mice).

Supplementary Figure 9. Photoactivation of eNpHR3.0 by 593 nm laser, but not a 405 nm laser, inhibits firing of GABA neurons.

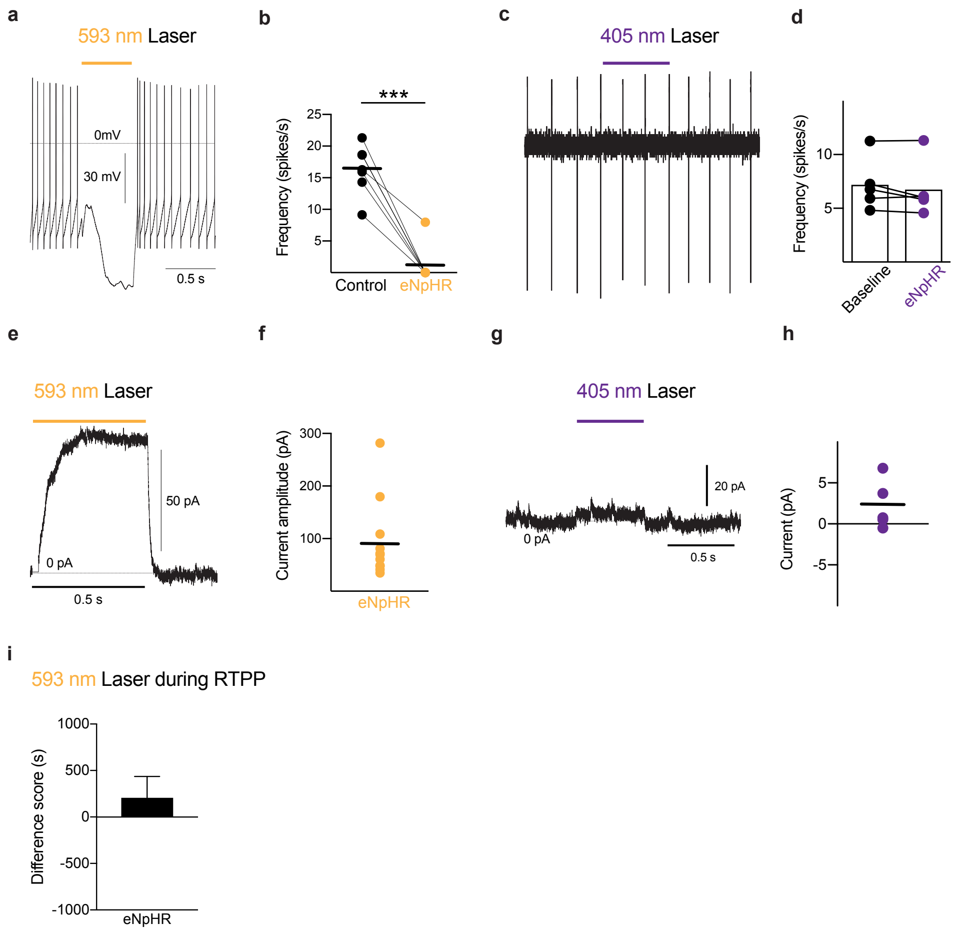

(a) Whole cell current-clamp recording from a VTA GABA neuron obtained from a Vgat-Cre mouse expressing eNpHR3.0 in GABA neurons. Photoactivation of eNpHR3.0 using a 593 nM laser prevents firing of action potentials in GABA neurons. **(b)** Summarized data showing the effect eNpHR3.0 (yellow) has on the firing of action potentials in GABA neurons (paired t-test $t_{(6)} = 8.141$, P = 0.0002, n =7 cells, 3 mice). **(c)** Cell-attached recording from a GABA neuron where 405 nm laser was applied with **(d)** accompanying summarized data (paired t-test $t_{(4)} = 1.562$, P = 0.19, n = 5 cells, 2 mice). **(e)** Whole-cell voltage clamp recording from a GABA neuron where activation of eNpHR3.0 with a 593 nM laser produces an outward current with **(f)** a summary of the currents generated (paired t-test $t_{(10)} = 4.01$, P = 0.002, n =11 cells, 3 mice). **(g)** A voltage-clamp recording from the same GABA neuron as (c) where activation of a 405 nm laser with equivalent light intensity does not change the firing frequency of GABA neurons. **(h)** Summary of the outward current produced by a 405 nm laser on GABA neurons (paired t-test $t_{(4)} = 1.523$, P = 0.20, n = 5 cells, 2 mice). **(i)** Summarized data demonstrating GABA neuron hyperpolarization does not produce rewarding affects in the RTPP behavior assay (paired t-test $t_{(6)} = 0.9036$, P = 0.4, n = 7 mice). ***: P < 0.001. Error bars indicate ± SEM.

Supplementary Figure 10. Astrocyte activation does not alter locomotor activity in real-time place preference or conditioned place preference assays.

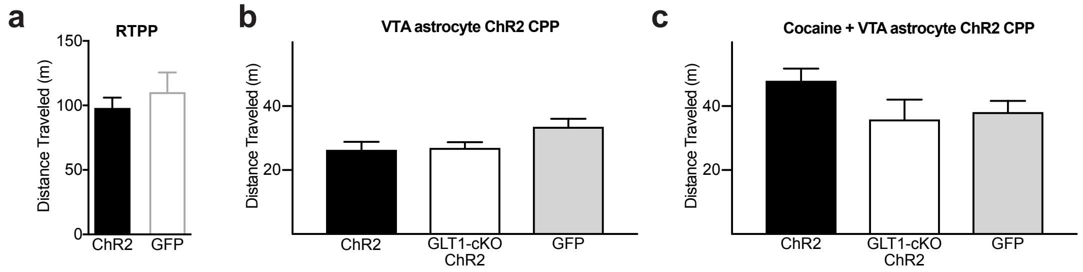

(a) There was no difference in the total distance traveled between mice expressing GFP or ChR2 in VTA astrocytes during a 30 minute RTPP session (unpaired t-test $t_{(15)} = 0.7177$, $P = 0.48$, $n = 8$ and $n = 9$ mice respectively). **(b)** Comparing the total distance traveled between mice expressing either ChR2, GLT-1 cKO with ChR2, or GFP in VTA astrocytes during laser activation (One-way ANOVA, $F_{(2,33)} = 2.203$, $P = 0.1264$). **(c)** Comparing the total distance traveled between mice expressing either ChR2, GLT-1 cKO with ChR2, or GFP in VTA astrocytes during cocaine + laser activation (One-way ANOVA, $F_{(2,27)} = 2.299$, $P = 0.1197$); Error bars indicate ± SEM.
