# Supplementary material for: Ventral tegmental area astrocytes orchestrate avoidance and approach behavior
Source: Nat Commun. 2019 Mar 29;10:1455. doi: 10.1038/s41467-019-09131-y (PMC6440962; doi:10.1038/s41467-019-09131-y)
Supplement: Supplementary file 2 — Description of Additional Supplementary Files [file 41467_2019_9131_MOESM2_ESM.pdf]

Description of Additional Supplementary Files

File Name: Supplementary Movie 1

Description: ChR2 stimulation on VTA astrocytes elicits avoidance. Mouse receives ChR2 photoactivation of VTA astrocytes (1Hz: 0.5 s pulse duration, 15-20 mW) when venturing to the top half of the field. Movie (4X speed) is 15 s of the 30 minute session.
