# Supplementary material for: Ventral tegmental area astrocytes orchestrate avoidance and approach behavior
Source: Nat Commun. 2019 Mar 29;10:1455. doi: 10.1038/s41467-019-09131-y (PMC6440962; doi:10.1038/s41467-019-09131-y)
Supplement: Supplementary file 4 — Reporting Summary [file 41467_2019_9131_MOESM4_ESM.pdf]

nature research

Corresponding author(s): Carlos A. Paladini

Last updated by author(s): Feb 21, 2019

Reporting Summary

Nature Research wishes to improve the reproducibility of the work that we publish. This form provides structure for consistency and transparency in reporting. For further information on Nature Research policies, see Authors & Referees and the Editorial Policy Checklist.

Statistics

For all statistical analyses, confirm that the following items are present in the figure legend, table legend, main text, or Methods section.

n/a	Confirmed	
☐	☒	The exact sample size (*n*) for each experimental group/condition, given as a discrete number and unit of measurement
☐	☒	A statement on whether measurements were taken from distinct samples or whether the same sample was measured repeatedly
☐	☒	The statistical test(s) used AND whether they are one- or two-sided<br>*Only common tests should be described solely by name; describe more complex techniques in the Methods section.*
☒	☐	A description of all covariates tested
☐	☒	A description of any assumptions or corrections, such as tests of normality and adjustment for multiple comparisons
☐	☒	A full description of the statistical parameters including central tendency (e.g. means) or other basic estimates (e.g. regression coefficient) AND variation (e.g. standard deviation) or associated estimates of uncertainty (e.g. confidence intervals)
☐	☒	For null hypothesis testing, the test statistic (e.g. *F*, *t*, *r*) with confidence intervals, effect sizes, degrees of freedom and *P* value noted<br>*Give P values as exact values whenever suitable.*
☒	☐	For Bayesian analysis, information on the choice of priors and Markov chain Monte Carlo settings
☒	☐	For hierarchical and complex designs, identification of the appropriate level for tests and full reporting of outcomes
☒	☐	Estimates of effect sizes (e.g. Cohen's *d*, Pearson's *r*), indicating how they were calculated

Our web collection on statistics for biologists contains articles on many of the points above.

Software and code

Policy information about availability of computer code

Data collection

Images were analyzed using ImageJ FIJI software (https://fiji.sc).
Flow cytometry was done using FlowJo software (https://www.flowjo.com)
Behavior data was collected using Anymaze (Stoelting) video tracking system (http://www.anymaze.co.uk).
Electrophysiology was collected and analyzedusing Axograph X (https://axograph.com)

Data analysis

Data analysis and statistics were performed using Prism software (https://www.graphpad.com/scientific-software/prism/).

For manuscripts utilizing custom algorithms or software that are central to the research but not yet described in published literature, software must be made available to editors/reviewers. We strongly encourage code deposition in a community repository (e.g. GitHub). See the Nature Research guidelines for submitting code & software for further information.

Data

Policy information about availability of data

All manuscripts must include a data availability statement. This statement should provide the following information, where applicable:

- Accession codes, unique identifiers, or web links for publicly available datasets
- A list of figures that have associated raw data
- A description of any restrictions on data availability

Data has been deposited in figshare: https://figshare.com/articles/ncomms-source-data_xlsx/7688894
Data is provided for main figures 1-8 and supplementary file figures 2-10.

Field-specific reporting

Please select the one below that is the best fit for your research. If you are not sure, read the appropriate sections before making your selection.

☒ Life sciences ☐ Behavioural & social sciences ☐ Ecological, evolutionary & environmental sciences

For a reference copy of the document with all sections, see nature.com/documents/nr-reporting-summary-flat.pdf

Life sciences study design

All studies must disclose on these points even when the disclosure is negative.

Sample size	Sample sizes were based on power analysis showing greater than 80 % power in all tests.
Data exclusions	A total of 3 mice were excluded from analysis for having virus and/or fiber optics outside of the VTA.<br>One mouse with a strong initial preference for one chamber in the CPP assay (defined as a mouse spending more than 75% of the time in either chamber) was discarded from analysis.
Replication	Behavioral tests were repeated in several cohorts and by different experimenters.
Randomization	Mice were selected at random for viral injections. Control mice were littermates with control injections.
Blinding	Electrophysiology experiments were performed with the recorder blind to the behavioral results. Behavioral tests were performed with the experimenter blind to the virus injection.

Reporting for specific materials, systems and methods

We require information from authors about some types of materials, experimental systems and methods used in many studies. Here, indicate whether each material, system or method listed is relevant to your study. If you are not sure if a list item applies to your research, read the appropriate section before selecting a response.

Materials & experimental systems

n/a	Involved in the study	
☐	☒	Antibodies
☒	☐	Eukaryotic cell lines
☒	☐	Palaeontology
☐	☒	Animals and other organisms
☒	☐	Human research participants
☒	☐	Clinical data

Methods

n/a	Involved in the study	
☒	☐	ChIP-seq
☐	☒	Flow cytometry
☒	☐	MRI-based neuroimaging

Antibodies

Antibodies used	Rabbit anti NeuN (1:1000, ABCAM), chicken anti tyrosine hydroxylase (1:500, AB9702), rabbit anti EAAT-2 (1:500, AB41621), mouse anti GABA (A0310)mouse anti- GFAP (1:1000, G3893), rabbit anti EEAT-2 (1:1000, AB41621) followed by secondary antibodies: donkey anti mouse Alexa 488 (1:1000, A21202), donkey anti rabbit Alexa 555 (1:1000, A31572),goat anti- rabbit Alexa 488 (1:1000, A11034) or Alexa 546 (1:1000, A11035), goat anti-chicken Alexa 488 (1:1000, A11039), Alexa 546 (1:1000, A11040) or 647 (1:1000, A21449)
Validation	Antibodies are validated by the commercial vendor using western blot, ICC/IF, IHC, flow cytometry, ELISA, ChIP, IP and peptide array.

Animals and other organisms

Policy information about studies involving animals; ARRIVE guidelines recommended for reporting animal research

Laboratory animals	Male and female C57BL/6J mice, Vgat-ires-Cre mice (016962 Stock) and GLT-1f/f (GLT-1flox/flox)39 mice were used. GLT-1f/f mice (Slc1A2tm1.1Pros ; MGI: 5752263) were obtained from the founder colony at Boston Children's Hospital.
Wild animals	Wild animals were not used.
Field-collected samples	This study did not involve samples collected from the field.

Ethics oversight	All procedures were approved by the University of Texas at San Antonio Institutional Animal care and Use Committees in accordance with the National Institutes of Health guidelines.

Note that full information on the approval of the study protocol must also be provided in the manuscript.

Flow Cytometry

Plots

Confirm that:

☒ The axis labels state the marker and fluorochrome used (e.g. CD4-FITC).

☒ The axis scales are clearly visible. Include numbers along axes only for bottom left plot of group (a 'group' is an analysis of identical markers).

☒ All plots are contour plots with outliers or pseudocolor plots.

☒ A numerical value for number of cells or percentage (with statistics) is provided.

Methodology

Sample preparation	The VTA was carefully sectioned from GLT-1f/f and cKO mice in 1X Hanks's Balanced Salt Solution (HBSS). The tissue was minced and incubated in pronase and repeatedly triturated every 20 minutes for 1 hour. DNASE (100 units/ml, Worthington) was added to the HBSS containing pronase solution to prevent cell aggregation. After 1 hour the cell suspension was diluted with 1X HBSS and centrifuged at 500g for 5 minutes. The pellet was resuspended in HBSS and filtered through a 100 µm filter. The cells were counted, and the filtrate was centrifuged and resuspended in Phosphate Buffer Saline.
Instrument	Cells were analyzed in a BD LSR II flow cytometer (http://www.bdbiosciences.com) equipped with three excitation lasers (488 nm, 555 nm and 633 nm)
Software	Analysis was done using FlowJo software (https://www.flowjo.com).
Cell population abundance	Cells were FACS analyzed for GLT-1 and GFAP. Cells were not sorted for purity and therefore there were no post sort fractions.
Gating strategy	All gates were determined based on unstained, single-color and fluorescence minus 1 GLT-1f/f of mouse brain cells.

☒ Tick this box to confirm that a figure exemplifying the gating strategy is provided in the Supplementary Information.
